# USLE-Based Assessment of Soil Erosion by Water in the Nyabarongo River Catchment, Rwanda

**DOI:** 10.3390/ijerph13080835

**Published:** 2016-08-20

**Authors:** Fidele Karamage, Chi Zhang, Alphonse Kayiranga, Hua Shao, Xia Fang, Felix Ndayisaba, Lamek Nahayo, Christophe Mupenzi, Guangjin Tian

**Affiliations:** 1 State Key Laboratory of Desert and Oasis Ecology, Xinjiang Institute of Ecology and Geography, Chinese Academy of Sciences, Urumqi 830011, China; fidelekaramage@yahoo.com (F.K.); kayiranga2020@yahoo.co.uk (A.K.); shaohua@ms.xjb.ac.cn (H.S.); sun@163.com (X.F.); davfelix@yahoo.fr (F.N.); lameknahayo@gmail.com (L.N.); mupenzic@gmail.com (C.M.); 2 University of Chinese Academy of Sciences, Beijing 100049, China; 3 Faculty of Environmental Studies, University of Lay Adventists of Kigali (UNILAK), P.O. 6392, Kigali, Rwanda; 4 School of Resources Environment Science and Engineering, Hubei University of Science and Technology, Xianning 437000, China; 5 State Key Laboratory of Water Environment Simulation, School of Environment, Beijing Normal University, Beijing 100875, China; tianguangjin@bnu.edu.cn

**Keywords:** soil erosion, water pollution, cropland, land-cover and land-use, USLE, GIS, remote sensing, Nyabarongo River Catchment, Rwanda

## Abstract

Soil erosion has become a serious problem in recent decades due to unhalted trends of unsustainable land use practices. Assessment of soil erosion is a prominent tool in planning and conservation of soil and water resource ecosystems. The Universal Soil Loss Equation (USLE) was applied to Nyabarongo River Catchment that drains about 8413.75 km^2^ (33%) of the total Rwanda coverage and a small part of the Southern Uganda (about 64.50 km^2^) using Geographic Information Systems (GIS) and Remote Sensing technologies. The estimated total annual actual soil loss was approximately estimated at 409 million tons with a mean erosion rate of 490 t·ha^−1^·y^−1^ (i.e., 32.67 mm·y^−1^). The cropland that occupied 74.85% of the total catchment presented a mean erosion rate of 618 t·ha^−1^·y^−1^ (i.e., 41.20 mm·y^−1^) and was responsible for 95.8% of total annual soil loss. Emergency soil erosion control is required with a priority accorded to cropland area of 173,244 ha, which is extremely exposed to actual soil erosion rate of 2222 t·ha^−1^·y^−1^ (i.e., 148.13 mm·y^−1^) and contributed to 96.2% of the total extreme soil loss in the catchment. According to this study, terracing cultivation method could reduce the current erosion rate in cropland areas by about 78%. Therefore, the present study suggests the catchment management by constructing check dams, terracing, agroforestry and reforestation of highly exposed areas as suitable measures for erosion and water pollution control within the Nyabarongo River Catchment and in other regions facing the same problems.

## 1. Introduction

The global freshwater is only 2.5% of total global water of which a little more than 1.2% is surface water [1]. However, every day, 2 million tons of sewage, industrial and agricultural waste are discharged into the world’s water [2]. Humans have increased the sediment transport by global rivers through soil erosion (by 2.3 ± 0.6 billion metric tons per year) [3]. Pollution can be so severe that the fresh water is no longer usable without incurring unacceptably high cleanup costs [4]. In addition, depending on the type and level of pollution, the water body may become also unsuitable for fishing, swimming, or even for aquatic animals to survive in [5].

Presently, land cover and land-use change is a critical world problem. There was a net decrease in global forest area of 1.7% between 1990 and 2005. Africa experienced net annual forest area losses of 1.1 million ha between 1990 and 2000 and 2.7 million ha between 2000 and 2005 [6]. In most of sub Saharan Africa, more than 50% of the populations rely on agriculture for their livelihood [7]. Just 25 years ago (in 1990), forest covered about 44% of Rwanda’s territory, while the cropland only occupied 28%. In 2015, more than 56% of the country’s land areas have been converted to croplands to meet the food demands, at the extent of massive deforestation [8]. However, these land conversions mainly for agricultural land use are associated with severe environmental problems including soil erosion by water and its water pollution [8]. Around 80% of the pollution in seas and oceans comes from land-based activities [9,10].

Lake Victoria, which drains the water from the Nyabarongo River through the Akagera River has been listed among the top 10 most highly polluted water bodies in the world [11]. This pollution is accelerated by intensive agriculture due to rapid population growth and industrialization in their riparian communities [12]. As a consequence, nutrients from erosion have caused the infestation of water hyacinth in Lake Victoria and the Akagera river basin [13,14].

In Rwanda, the actual soil erosion by water was estimated at approximately 595 million tons per year with a mean soil erosion rate of 250 t·ha^−1^·y^−1^ [8]. Erosion causes soil nutrients losses estimated at 945,200 t of organic materials, 41,210 t of nitrogen, 200 t of phosphorus and 3055 t of potash annually [15]. Soil erosion caused by land reclamation seriously threatens the water quality in Rwanda [16,17,18], where a households-based survey indicated its contribution to about 61.8% for water pollution within the Nyabarongo River system [19]. The water in the Nyabarongo River system is polluted as far as physical parameters are concerned with high Turbidity of 737.28 ± 571.03 Nephelometric Turbidity Units (NTU), Nitrate–Nitrogen (NO_3_–N) of 28.79 ± 20.94 mg/L compared to 1 NTU and 11 mg/L, respectively, set as the standard limits by the WHO Drinking Water Guideline [20]. Previous studies have focused on the analysis of water quality in Nyabarongo River and quasi-unanimously pointed out soil erosion as the main causal agent. However, few attempts have dealt with elucidating the causes of soil erosion and quantitatively assessing the soil erosion status in the Nyabarongo River Catchment; however, such information is critical for the effective management of the catchment. The objectives of this study are, therefore, (1) to delineate the extent of the Nyabarongo River Catchment; (2) assess the causes of soil erosion in the catchment in the form of Land Cover and Land use (LCLU) and geomorphology and (3) estimate the actual soil erosion rate in the catchment. To achieve the research objectives, this study used the Universal Soil Loss Equation (USLE) model developed by the United States Department of Agriculture (USDA) to predict the longtime average annual inter-rill and rill soil loss under various effects such as rainfall, soil types, topography, and land cover type and land use [21].

## 2. Materials and Methods

### 2.1. Description of the Study Area

**Figure 1:**
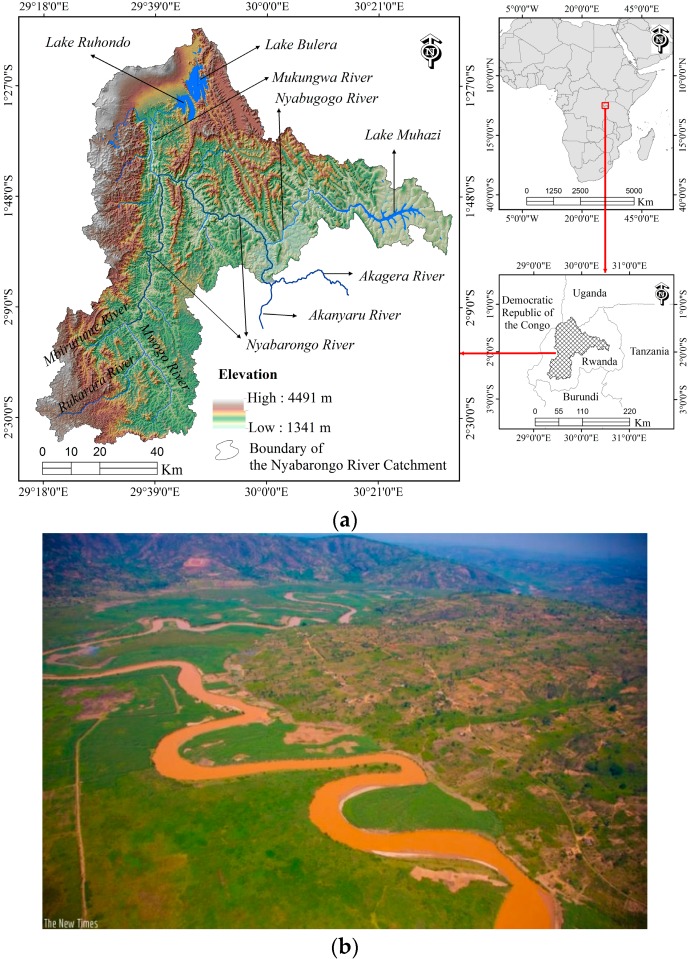
(**a**) Location map of the Nyabarongo River Catchment; and (**b**) an aerial view of the Nyabarongo River, with the water looking muddy brown due to pollution [26].

The Nyabarongo River (Figure 1) has an estimated length of 151.5 km and drains a total area of 8478.24 km^2^ (8413.75 km^2^ or 33% of the total Rwanda coverage plus 64.50 km^2^, a small part of the Southern Uganda). The catchment is mostly formed by a mountainous terrain with a mean slope of 30% and elevation ranging from 1341 m to 4491 m. The underlying geology consists of 59.3% Acrisols, 19.2% Regosols, 9.2% Andosols, 6.7% Ferralsols, 2.8% Cambisols, 0.8% Histosols, 0.4% Gleysols, and other 1.5% is covered by water [22]. This catchment experiences a tropical climate with two rainfall seasons a year, March to May and September to December [23], an annual mean temperature of 17 °C and mean precipitation of 1231 mm/y [24]. Precipitation increases with elevation, which varies from about 864 mm in the central plateau across the Lake Muhazi and Kigali City to 2258 mm in the mountain ranges over Nyungwe forest and volcano areas. The Nyabarongo River starts from the confluence of Mbirurume and Mwogo rivers and ends up at its confluence with the Akanyaru River, emptying into the Akagera River, one of the largest rivers that drains into Lake Victoria [25]. The Nyabarongo River serves as a tributary of the Nile River and provides goods and services such as a source of drinking water, irrigation and fishery to the Rwandese communities; however, it is currently facing heavy pollution from mining, encroachment, landslides, unsustainable agriculture and domestic and industrial wastes [26].

### 2.2. Delineation of the Catchment

The Nyabarongo River Catchment (Figure 1a) was delineated from the Advanced Space borne Thermal Emission and Reflection Radiometer (ASTER) Global Digital Elevation Model (GDEM) version 2 (30 m resolution) acquired from the United States Geological Survey (U.S.G.S.) EarthExplorer (EE) database [27], using the hydrology toolset of the ArcGIS software version 10.2 (Environment Systems Research Institute (Esri) Inc., Redlands, CA, USA).

### 2.3. Preparation of LCLU Map for the Nyabarongo River Catchment

A land cover and land use (LCLU) map of the Nyabarongo River Catchment was developed from four Landsat 8 images (path; row: 173/61; 173/62 acquired on 21 September 2015 and 172/61; 172/62 acquired on 12 July 2015) from the U.S.G.S. global visualization tool [28]. ENVI software version 5.2 (Exelis Visual Information Solutions, Inc., a subsidiary of Harris Corporation, Boulder, CO, USA) was used to develop the Land Cover and Land Use map of the Nyabarongo River Catchment. Firstly, these images were radiometrically corrected and the cloud-shadows were masked and the gap filling algorithm was used to obtain a cloud-free image [29]. The supervised maximum likelihood classification method [30] used in this study is the most common supervised classification method used with remote sensing image data, and it was found to be more applicable and reliable for the satellite image classification purposes [30,31]. Six LCLU classes (Settlement, Cropland, Forestland, Grassland, Wetland and Water Bodies) within the study area were identified based on the U.S.G.S. classification system level 1 [32] and are enough to analyze the influence of land use on soil erosion for our case study. Referring to the accuracy assessment methods [33,34,35], 60 points were randomly selected on the original Landsat image for each land cover category and, thereafter, the overlay of classified image on the Google Earth program (Google Inc., Amphitheatre Parkway Mountain View, San Jose, CA, USA) was applied for accuracy verification.

**Figure 2:**
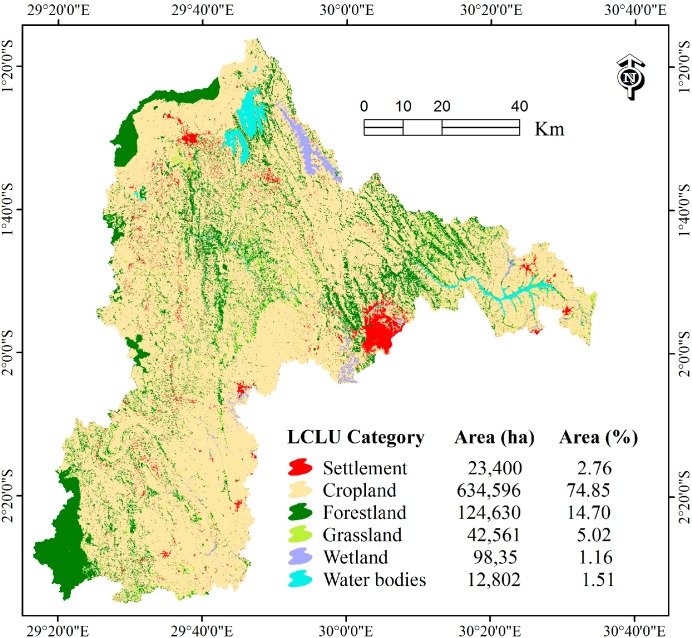
Land Cover and Land Use (LCLU) for the Nyabarongo River Catchment in 2015.

The overall accuracy for the LCLU classification of the Nyabarongo River Catchment was 91% (Table 1). This accuracy is acceptable compared to the recommended overall classification accuracy of at least 85% [32,36,37] and 70% for each land use category accuracy [36]. The LCLU map for the study area indicates that cropland comprised about 75% of the total catchment (Figure 2).

### 2.4. Development of the USLE Factors

The ArcGIS software has become a prominent tool for the USLE (Equation (1)) modeling. This approach has been used successfully in various studies to assess soil loss and its planning management [8,38,39,40,41,42,43,44]. Using the nearest-neighbor method, all the datasets utilized in this study were resampled to the same spatial resolution of 30 × 30 m and reprojected to the World Geodetic System (WGS) 1984_Universal Traverse Mercator (UTM) zone 35 south because they were acquired from different sources with different spatial resolutions.

A = R × K × LS × C × P
(1)

where A: average annual soil loss per unit area (t·ha^−1^·y^−1^); R: rainfall-runoff erosivity factor (MJ·mm·ha^−1^·h^−1^·y^−1^); K: erodibility factor (t·h·MJ^−1^·mm^−1^); LS: slope length (L) and the slope steepness (S) factor; C: cover and management factor; and P is the support and conservation practices factor [45].

#### 2.4.1. Rainfall Erosivity Factor (R)

Rainfall erosivity contributes to about 80% of soil loss [46] and has significant impacts on soil erosion [47]. Because of the paucity of R measurement data worldwide [48] and the lack of rain gauged meteorological data in the study area [8], we have been constrained to use R factor derived from the Global raster dataset provided by the Global Land Degradation Information System (GLADIS) database provided by the Food Agriculture Organization (FAO) [49]. Given that in tropical areas the transformation of the Fournier index into the R factor works less well, R factor has been generated using an alternative index related to annual rainfall (Equation 2) developed by Lo et al. [48,50]:

(2) R=[38.46+(3.48×P)]

where *P* is the Average annual rainfalls.

**Figure 3:**
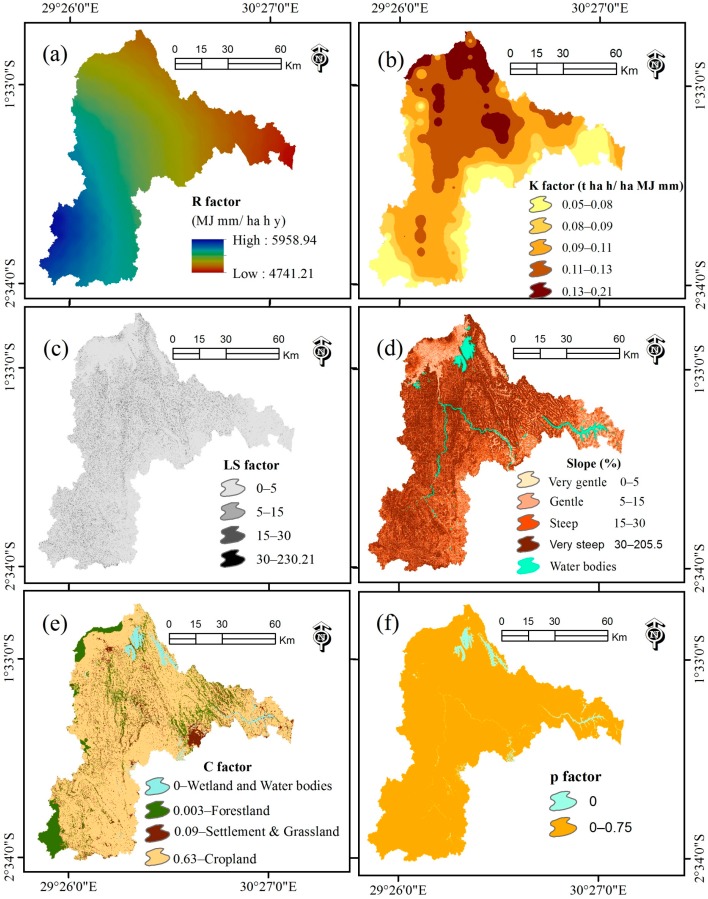
Maps of the Universal Soil Loss Equation (USLE) factors for the Nyabarongo River Catchment: (**a**) rainfall erosivity; (**b**) soil erodibility; (**c**) slope length and slope steepness; (**d**) the Slope angle; (**e**) land cover management; (**f**) conservation support practice.

The R factor for Nyabarongo River Catchment has the value ranging from 4741.21 MJ·mm·ha^−1^·h^−1^·y^−1^ to 5958.94 MJ·mm·ha^−1^·h^−1^·y^−1^ (Figure 3a).

#### 2.4.2. Soil Erodibility Factor (K)

The K factor (Equation (3)) is a quantitative value that is experimentally determined taking into consideration the soil texture and structure, the organic matter content and the permeability [21]:

(3) $$K=2.1\times 10^{-6}\times M^{1.14}\times \left(12-OM\right)+0.0325\times \left(P-2\right)+0.025\times \left(S-3\right)$$

where *M*: (% silt + % very fine sand) (100% clay); *OM* = percentage of organic matter; *P*: permeability class; and *S*: structure class.

Erosion models often use secondary data available in a geographic information system as an alternative approach mostly when assessing soil erosion for a large area because the measurement of soil erosion is expensive and time consuming due to extensive laboratory analysis of samples of different soil profiles [8,42,51]. Therefore, the K factor used in this study with values ranging from 0.05 t·h·MJ^−1^·mm^−1^ to 0.21 t·h·MJ^−1^·mm^−1^ (Figure 3b) was derived from Global raster datasets in grid format as provided by the Global Land Degradation Information System (GLADIS) database of the Food Agriculture Organization (FAO) [49]. This project obtained the global K factor dataset by applying the soil erodibility classification for water erosion FAO 1984 to the soil units of the soil map of the world, both FAO 1974 and 1990 legends. Furthermore, the reference factor was adjusted for topsoil texture characteristics and soil phase characteristics influencing resistance to rainfall impact such as surface stoniness or influencing soil permeability such as lithic, plastic, anthraquic soil phases or other impermeable layers. Saline and sodic soil phases reduce soil permeability of subsoil and therefore indirectly increase soil erodibility. The modifiers (topsoil texture, protection impact and reduced permeability) have consecutively been applied to the reference soil erodibility factor of each soil unit and a weighted average for each soil mapping unit has been calculated [50].

#### 2.4.3. Slope Length and Steepness Factor (LS)

The slope length factor (L) represents the effect of slope length on erosion, and the slope steepness factor (S) reflects the influence of slope gradient on erosion [21]. The LS factor (Figure 3c) for this study was estimated from the ASTER GDEM of 30 m resolution [27] using Equation (4) [52] computed with the Raster Calculator tool from the Spatial Analyst extension of ArcMap (Environment Systems Research Institute (Esri) Inc., Redlands, CA, USA). The Flow Accumulation tool available in the Spatial Analyst Hydrology toolset of ArcMap was used to calculate the Flow Accumulation grid. The Grid slope in percentage was calculated with the Slope tool in the Spatial Analyst Surface toolset of ArcMap:

(4) $$LS={\left[\frac{Q_{a}M}{22.13}\right]}^{y}\times \left(0.065+0.045\times S_{g}+0.0065\times S_{g}^{2}\right)$$

where LS: topographical factor; $Q_{a}$: Flow Accumulation grid; $S_{g}$: Grid slope in percentage; *M*: Grid size (Vertical length times horizontal length), *y*: a constant dependent on the value of the slope gradient: 0.5 if the slope is greater than 4.5%, 0.4 on slopes of 3% to 4.5%, 0.3 on slopes of 1% to 3%, and 0.2 on slopes less than 1%. Therefore, in our study a constant (*y*) of 0.5 was used in Equation (4) due to the mean slope of 30% observed for the Nyabarongo River Catchment. The ASTER GDEM was chosen to be used in this study due to its finer spatial resolution that closely matches that of the Geo-referenced Landsat images and at comparable accuracies [29,53] and it was found to be reliable for *LS* factor generation [44]. Accuracies for this global product were estimated with 20 m at 95% confidence for vertical data and 30 m at 95% confidence in horizontal data [54]. The *LS* factor (Figure 3c) has values ranging from 0 to 230.21. This range indicates an area with very steep slopes where 40% of the land catchment has very steep slopes ranging from 30% to 205.5% gradient (Figure 3d). Generally, the range of slope values in degrees is from 0 to 90. For percent rise, the range is 0 for near infinity. A flat surface is 0%, a 45 degree surface is 100%, and as the surface becomes more vertical, the percentage rise becomes increasingly larger [55]. This expresses that the cells with slopes >100% in the Nyabarongo River Catchment are for the extremely steep surface >45 degrees gradient. 

#### 2.4.4. Cover Management Factor (C)

The C factor reflects the effects of cropping and management practices on soil erosion rates in agricultural lands and the effects of vegetation canopy and ground covers on reducing the soil erosion in forested regions [45]. Wet seasons associated with the low vegetation coverage are most frequently the optimum period for estimating an annual soil loss whenever land is highly exposed to soil erosion risk [56]. The normalized vegetation index (NDVI) is the source of C factor values. The accuracy of these values can be maximized taking into account their quantitative assessment such as cover type, arboreal height, percentage of tree cover and shrubs and thickness of grass cover and humus [57]. However, in some places, mostly in tropical areas including the area under investigation in this study, the abundance of thick clouds [29] remains a challenge for quality remote sensing data. In spite of these challenges, the present study established the C factor map (Figure 3e) by attributing representative C factor values recommended by Wischmeier and Smith (1978) [21] to LCLU classes; 0, 0.003, 0.09, and 0.63 for the wetland, forest, settlement and grassland, and cropland, respectively, in accordance with the methodology adopted by a number of similar studies [8,40,52,58].

#### 2.4.5. Support Practice Factor (P)

The P factor plays an important role in the form of conservation practices [44]. According to the value of the P factor observed over Rwanda [50], the current study established the P factor map with 0 value for water and wetland and 0.75 for the rest of the LCLU classes (Figure 3f).

The soil erosion map was overlaid with the slope map (%) with four slope angle classes (<5% as very gentle to flat, 5% to 15% as gentle, 15% to 30% as steep and >30% as very steep) [59] to assess the influence of the slope steepness on the soil erosion rates. To assess the potential impact of terracing cultivation method, the P factor was estimated based on the slope and terracing cultivation method (Table 2) [60].

**Figure 4:**
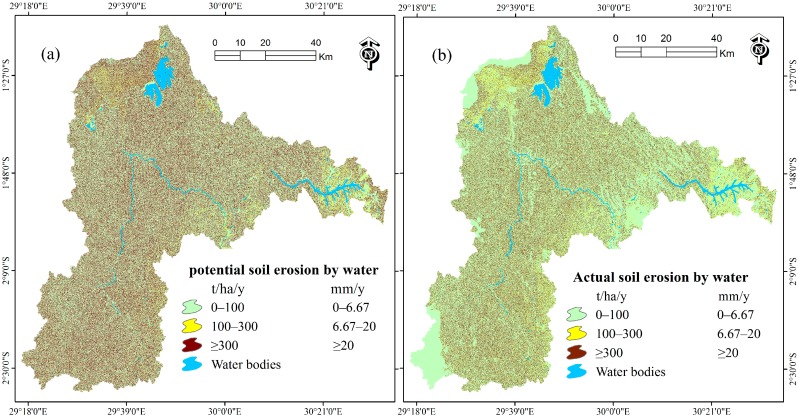
Maps of the Nyabarongo River Catchment: (**a**) potential soil erosion; and (**b**) actual soil erosion, 2015.

As recommended by Wischmeier and Smith [21], to identify the areas of greatest vulnerability in this study, a potential soil erosion map (Figure 4a) that considers only the natural factors if a field were continuously in fallow conditions and the actual soil erosion map (Figure 4b) that considers both natural and LCLU management practices have been generated by the multiplication of the R, K, LS factor maps and the R, K, LS, C, P factor maps respectively using the raster calculator function tool of the ArcGIS. Actual soil loss from the cropped field is usually much less than the potential soil loss depends on the particular combination of cover, crop sequence, and management practices [21]. The statistics of soil erosion rates presented in Table 3, Table 4, Table 5, Table 6, Table 7, Table 8 and Table 9 were computed using the Zonal Statistics as Table Tool available in the Spatial Analyst Zonal Toolset of the ArcGIS software version 10.2 (Environment Systems Research Institute (Esri) Inc., Redlands, CA, USA).

## 3. Results

Soil erosion maps are presented with double units where 15 t·ha^−1^·y^−1^ = 1 mm·y^−1^ according to FAO recommendation [57,61]. Erosion rates were classified into three categories, notably Moderate (0 t·ha^−1^·y^−1^–100 t·ha^−1^·y^−1^), High (100 t·ha^−1^·y^−1^–300 t·ha^−1^·y^−1^) and Extreme (≥ 300 t·ha^−1^·y^−1^) [58]. The results from natural soil erosion factors (rainfall erosivity, soil erodibility and slope length and slope steepness) indicate that the Nyabarongo river Catchment is naturally vulnerable to soil erosion by water with a rate of 1397 t·ha^−1^·y^−1^ or 93.13 mm·y^−1^. The areas of greatest vulnerability with extreme potential erosion rate of 4487 t·ha^−1^·y^−1^ or 299.13 mm·y^−1^ comprised 31% of the catchment and contributed up to 99.54% of the total annual soil loss (Figure 4a and Table 3). When taking into accounts land cover and land use (C) and support practice (P) factors in 2015, the catchment is associated with an actual soil erosion rate of 490 t·ha^−1^·y^−1^ or 32.67 mm·y^−1^ and 22% of the catchment is exposed to extreme soil erosion of 2178 t·ha^−1^·y^−1^ or 145.2 mm·y^−1^ that contributed 97.8% of the total annual soil loss (Figure 4b and Table 4).

LCLU is represented by the values of C factor. The cropland that is associated with the highest C factor value of 0.63 comprised 76% of the total land catchment and is exposed to the highest estimated mean erosion rate of 618 t·ha^−1^·y^−1^ compared to other land use classes. 95.8% of the total soil loss was observed in cropland area (Table 5).

Agricultural land use occupied a big portion of 94.3% in the extreme erosion areas (≥300 t·ha^−1^·y^−1^) and accounted for 96.2% of the soil loss in these extreme erosion areas (Table 6).

The catchment area was classified into four categories according to the slope angle: Very Gentle to Flat, Gentle, Steep and Very Steep [59]. The very steep slope area with a slope angle >30% occupied 44% of the land area within the catchment and is responsible for 73.5% of the total soil loss and is associated with high mean soil erosion rate of 819 t·ha^−1^·y^−1^ (i.e., 54.6 mm·y^−1^) compared to the other slope angle classes (Table 7).

## 4. Discussion

Extremely high population density in Rwanda coupled with heavy dependency on agriculture of 83.4% [15] exerts enormous pressure on ecosystems and natural forests [62]. Watersheds upstream and unplanned occupation of land result in severe erosion, causing a serious soil degradation [8,63,64]. Due to the dominant agricultural land use of 74.85% within the Nyabarongo River Catchment in 2015, the catchment exposed to mean soil erosion rate of 490 t·ha^−1^·y^−1^ or 32.67 mm·y^−1^ (Figure 4b and Table 4), an erosion rate that can occur in cropland areas on steep slopes and heavy precipitation as previously revealed by Karamage et al. while estimating soil erosion risk in Rwanda with a mean erosion rate (421 t·ha^−1^·y^−1^ or 28.07 mm·y^−1^) for the cropland cell [8].

The moderate and high actual erosion rates were estimated over approximately 78% of the catchment area excluding water bodies (Table 4). The findings of this study are congruent with the early findings that estimated the erosion rate of ≥300 t·ha^−1^·y^−1^ for other areas in the tropics [58,65]. Furthermore, the rest of the catchment area (22%) is subjected to an extreme soil erosion rate of 2178 t·ha^−1^·y^−1^ (Table 4). The majority of the upland soils in the humid and subhumid tropics is grouped as low activity clay (LAC) soils characterized by fragile soils due to its low Effective Cation Exchange Capacity (ECEC) of ≤16 meq/100 g clay in the subsoil [66,67]. Extreme soil erosion rates occurring in Rwanda including the Nyabarongo River Catchment are associated with very fragile soils derived from physico-chemical alteration of schistose, quartzite, gneissic, granite and volcanic rocks [68], steep slopes and high rainfall intensity as previously discussed while estimating the soil erosion in Nethravathi basin located in the middle region of Western Ghats, western India where soil erosion rate was ranging from 0 t·ha^−1^·y^−1^ to 1,907,287 t·ha^−1^·y^−1^ [52].

As previously indicated by various researches [8,43,50,52,58,59], clearance of natural biomass for agricultural land use on steep slopes is the main instigator of soil erosion. This is in agreement with the findings of this study where the cropland alone, occupying 76% of the land area within the catchment, was associated with a high erosion rate of 618 t·ha^−1^·y^−1^ or 41.20 mm·y^−1^ and comprised about 95.8% of the soil loss within the catchment (Table 5). In this study, the USLE model revealed that the slope gradient plays a significant role on soil erosion within the catchment of Nyabarongo River, where the soil erosion rate increased in compatibility with the slope angle. The area with a very steep slope (>30%) was associated with a mean soil erosion rate of 819 t·ha^−1^·y^−1^ or 54.6 mm·y^−1^ and accounted for 73.5% of the total soil loss within the catchment (Table 7).

The present study found that the cropland largely extended to regions with extreme soil erosion, represented a big portion (94.3%) of the areas linked with extreme erosion rates, and was responsible for 96.2% of soil loss in these areas (Table 6). A percentage nearly equivalent to the percentage of extreme soil loss (96.9%) estimated for the agricultural land use in the Rio Lempa, Central America, indicating that agricultural land use is an important contributing factor in nearly all areas with extreme and high erosion rates [58]. 

The government of Rwanda, through the Organic Law number 04/2005, has established protection measures to protect river shores and wetlands in general; this has obliged the removal of agricultural crops in the 10 riparian meters and specific crops to be put on those 10 m. If this law is followed, it would lead to the improvement of water quality without water hyacinths and would be favorable for transport activities, biodiversity conservation and tourism. On the river shores, the natural vegetation will constitute a good habitat for reproduction of fish and increase its productivity. Agroforestry, reeds and bamboos on river shores will constitute another source of food and income for the owners. The survey showed that 63.8% households knew the Organic Law No. 04/2005 and among the total 359 households surveyed, 75.2% (270 households) agreed that the mean household’s maximum willingness to pay for the protection of Nyabarongo River system was 486.4 Rwandan francs (RWF) per household per month over the proposed five years (US$1 = RWF607) [19]. However, as indicated by this study, failure of extreme erosion control within the entire catchment rather than considering the buffer zone alone of the river might be the cause of high pollution due to soil sediments from various watersheds of the Nyabarongo River Catchment. Radical terraces that fight soil erosion have also had the potential to increase farm productivity and alleviate poverty. However, many difficulties such as sticky soils that are hard to work on, lack of equipment (i.e., farm tractors), steep slopes, lack of financial means and qualified supervisors constitute a monumental challenge to achieving the aims of radical terraces in Rwanda [15]. The water pollution is associated with degradation of natural biomasses where 14.1% of the country of Rwanda vegetation is degraded, from slight (7.5%) to substantial (6.6%) deterioration. A recent study on vegetation dynamics in Rwanda revealed that several types of vegetation were seriously endangered: the mosaic grassland/forest or shrubland was severely degraded, followed by sparse vegetation, grassland or woody vegetation regularly flooded on water logged soil [69].

**Figure 5:**
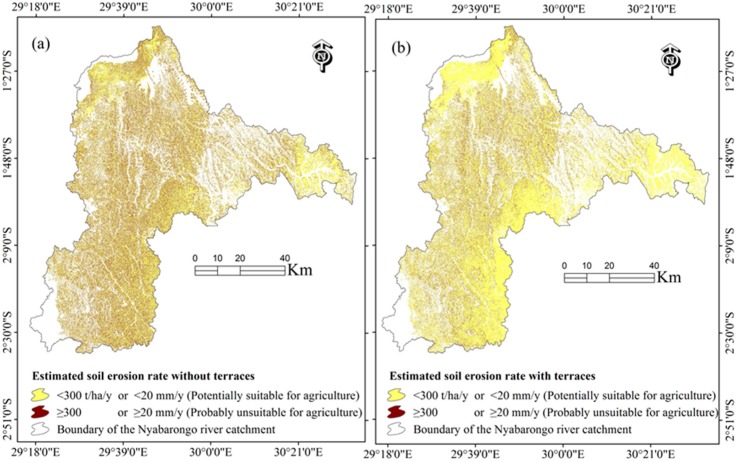
Maps of the agricultural land use suitability for the 2015 cropland; (**a**) agricultural land use suitability without terraces or with minor support practice (P = 0.75); and (**b**) agricultural land use suitability if terraces were applied on the cropland area.

Construction of Check dams, reservoirs and terraces might be suitable for sustainable soil erosion control measures within the Nyabarongo River Catchment based on the well-established importance of Check dam systems and natural vegetation rehabilitation [70], reservoirs that reduced the flux of sediment reaching the world’s coasts (by 1.4 ± 0.3 billion metric tons per year) and where 100 billion metric tons of sediment and 1 billion to 3 billion metric tons of carbon are now sequestered in reservoirs within the past 50 years [3], and terraces [8,41,45,60,71,72,73] on soil erosion mitigation reduction. Terracing has shown to be the best land conservation support practice when compared, for example, with strip-cropping and contouring cultivation methods [60]. The current study indicated that terraces could reduce the current erosion rate in cropland areas by about 78%, a reduction of mean soil erosion rate from 618 t·ha^−1^·y^−1^ to 134 t·ha^−1^·y^−1^ or 41.20 mm·y^−1^ to 8.93 mm·y^−1^ (Table 8 and Table 9, and Figure 5).

Potentially suitable areas comprised actual erosion rate of <300 t·ha^−1^·y^−1^ or <20 mm·y^−1^, while probably unsuitable areas are characterized by erosion rate of ≥300 t·ha^−1^·y^−1^ or ≥20 mm·y^−1^ under C factor value of 0.63 for agricultural land use [58].

## 5. Conclusions

The present study assessed soil erosion by water in the Nyabarongo River Catchment in Rwanda. The USLE model and GIS datasets were employed. The results indicated that the Nyabarongo River Catchment is extremely exposed to soil erosion by water due to unmanaged intensive cropland expansion over steep slopes that cleared a vast part of biomass cover by 76% of the land catchment in an area predominated by high rainfall intensity and fragile soil with high sensitivity to erosion. Consequently, an estimated 409 million tons of soils are lost every year in the catchment and pollute the water of Nyabarongo River. This study suggests the emergency need for water and soil conservation practices such as terracing cultivation methods, agroforestry, establishing biomass cover mostly on the land with very steep slopes, and construction of Check dams which could reduce the current cropland soil erosion and sediment loads and improve the water quality of the Nyabarongo River. Although this study identified the situation of land use and their related soil erosion rates, further research is suggested for this area such as a feasibility analysis of terracing implementation and a suitability analysis of Check dam locations within different watersheds of the Nyabarongo River Catchments.

## Figures and Tables

**Table 1:** Confusion Matrix for 2015 Land Cover and Land Use (LCLU) for the Nyabarongo River Catchment.

	1	2	3	4	5	6	∑	User Accuracy	Commission Error
1.Settlement	**53**	5	0	0	0	0	58	91%	9%
2.Cropland	7	**53**	0	3	0	0	63	84%	16%
3.Forestland	0	0	**53**	2	1	0	56	95%	5%
4.Grassland	0	2	7	**52**	2	0	63	83%	17%
5.Wetland	0	0	0	2	**57**	0	59	97%	3%
6.Water Bodies	0	0	0	0	1	**60**	61	98%	2%
∑	60	60	60	59	61	60	**360**	–	–
Producer Accuracy	88%	88%	88%	88%	93%	100%	–	–	–
Omission Error	12%	12%	12%	12%	7%	0%	–	–	–
Overall Accuracy	91%	–	–	–	–	–	–	–	–
Kappa	89%	–	–	–	–	–	–	–	–

**Table 2:** Support Practice Factor (P) for terracing.

Slope (%)	0–7	7–11.3	11.3–17.6	17.6–26.8	>26.8
P Factor	0.1	0.12	0.16	0.18	0.2

**Table 3:** Estimated potential soil erosion rates in the entire Nyabarongo River Catchment excluding water bodies (Figure 4a).

Soil Erosion Class (t·ha^−1^·y^−1^)	Area (ha)	Area (%)	Mean Erosion (t·ha^−1^·y^−1^)	Annual Soil Loss (t)	Annual Soil Loss (%)
Moderate (0–100)	551,114	66	0.6 ± 7	350,050	0.03
High (100–300)	25,051	3	200 ± 57	5,017,380	0.43
Extreme (≥300)	258,857	31	4487 ± 5310	1,161,465,050	99.54
Entire Catchment	835,022	100	1397 ± 3611	1,166,832,480	100

**Table 4:** Estimated actual soil erosion rates in the entire Nyabarongo River Catchment excluding water bodies, 2015 (Figure 4b).

Soil Erosion Class (t·ha^−1^·y^−1^)	Area (ha)	Area (%)	Mean Erosion (t·ha^−1^·y^−1^)	Annual Soil Loss (t)	Annual Soil Loss (%)
Moderate (0–100)	609,566	73	2 ± 12	1,227,482	0.3
High (100–300)	41,751	5	186 ± 58	7,774,055	1.9
Extreme (≥300)	183,705	22	2178 ± 2327	400,159,243	97.8
Entire Catchment	835,022	100	490 ± 1413	409,160,780	100

**Table 5:** LCLU 2015 and Estimated soil erosion rates in the entire Nyabarongo River Catchment excluding water bodies.

LCLU Class	Area (ha)	Area (%)	Mean Erosion (t·ha^−1^·y^−1^)	Annual Soil Loss (t)	Annual Soil Loss (%)
Settlement	23,400	2.8	105 ± 493	2,454,965	0.6
Cropland	634,596	76.0	618 ± 1569	391,976,027	95.8
Forestland	124,630	14.9	49 ± 508	6,137,412	1.5
Grassland	42,561	5.1	202 ± 744	8,592,376	2.1
Wetland	9835	1.2	0	0	0
Entire Catchment	835,022	100	490 ± 1413	409,160,780	100

**Table 6:** LCLU 2015 and estimated extreme soil erosion rates in the Nyabarongo River Catchment.

LCLU Category	Area (ha)	Area (%)	Mean Erosion (t·ha^−1^·y^−1^)	Annual Soil Loss (t)	Annual Soil Loss (%)
Settlement	1722	0.9	1162 ± 1445	2,000,796	0.5
Cropland	173,244	94.3	2222 ± 2340	384,953,192	96.2
Forestland	2066	1.1	2717 ± 2820	5,602,229	1.4
Grassland	6673	3.6	1139 ± 1556	7,603,026	1.9
Wetland	0	0	0	0	0
Extreme Erosion	183,705	100	2178 ± 2327	400,159,243	100

**Table 7:** Slope (%) and Estimated soil erosion rates in the entire Nyabarongo River Catchment excluding water bodies.

Description	Slope Angle (%)	Area (ha)	Area (%)	Mean Erosion (t·ha^−1^·y^−1^)	Annual Soil Loss (t)	Annual Soil Loss (%)
Very Gentle to Flat	<5	41,751	5	49 ± 39	2,045,804	0.5
Gentle	5–15	167,004	20	113 ± 169	18,821,396	4.6
Steep	>15–30	258,857	31	338 ± 563	87,560,407	21.4
Very Steep	>30	367,410	44	819 ± 2015	300,733,173	73.5
Entire Catchment	0–205.5	835,022	100	490 ± 1413	409,160,780	100

**Table 8:** Estimated actual soil erosion rates and Slope (%) for cropland cell (C factor = 0.63) of the Nyabarongo River Catchment and minor support practice of 0.75 (Figure 5a).

Agricultural Land Use Suitability	Erosion Rates (t·ha^−1^·y^−1^)	Area (ha)	Area (%)	Mean Erosion (t·ha^−1^·y^−1^)	Annual Soil Loss (t)	Annual Soil Loss (%)	Mean Slope (%)
Suitable	<300	463,255	73	15 ± 53	7,022,835	2	28 ± 19
Unsuitable	≥300	173,244	27	2222 ± 2340	384,953,192	98	29 ± 15
Cropland Cell	–	634,596	100	618 ± 1569	391,976,027	100	29 ± 18

**Table 9:** Estimated actual soil erosion rates and slope (%) for cropland cell (C factor = 0.63) within the Nyabarongo River Catchment and assumed terracing cultivation method as a sustainable support practice factor (Table 2 and Figure 5b).

Agricultural Land Use Suitability	Erosion Rates (t·ha^−1^·y^−1^)	Area (ha)	Area (%)	Mean Erosion (t·ha^−1^·y^−1^)	Annual Erosion (t)	Annual Erosion (%)	Mean Slope (%)
Suitable	<300	545,753	86	12 ± 59	6,549,036	8	27 ± 18
Unsuitable	≥300	88,843	14	885 ± 746	78,588,369	92	40 ± 13
Cropland Cell	–	634,596	100	134 ± 411	85,137,405	100	29 ± 18

## Abbreviations

MJ	Megajoule
milliequivalents	meq
US$	U.S. dollar
RWF	Rwandan Franc
